# The Impact of the New WHO Antiretroviral Treatment Guidelines on HIV Epidemic Dynamics and Cost in South Africa

**DOI:** 10.1371/journal.pone.0021919

**Published:** 2011-07-20

**Authors:** Jan A. C. Hontelez, Sake J. de Vlas, Frank Tanser, Roel Bakker, Till Bärnighausen, Marie-Louise Newell, Rob Baltussen, Mark N. Lurie

**Affiliations:** 1 Department of Public Health, Erasmus MC, University Medical Centre Rotterdam, Rotterdam, The Netherlands; 2 Africa Centre for Health and Population Studies, University of KwaZulu-Natal, Mtubatuba, South Africa; 3 Department of Primary and Community Care, Radboud University Nijmegen Medical Centre, Nijmegen, The Netherlands; 4 Department of Global Health and Population, Harvard School of Public Health, Boston, Massachusetts, United States of America; 5 Department of Epidemiology and the International Health Institute, Warren Alpert Medical School, Brown University, Providence, Rhode Island, United States of America; University of Cape Town, South Africa

## Abstract

**Background:**

Since November 2009, WHO recommends that adults infected with HIV should initiate antiretroviral therapy (ART) at CD4+ cell counts of ≤350 cells/µl rather than ≤200 cells/µl. South Africa decided to adopt this strategy for pregnant and TB co-infected patients only. We estimated the impact of fully adopting the new WHO guidelines on HIV epidemic dynamics and associated costs.

**Methods and Finding:**

We used an established model of the transmission and control of HIV in specified sexual networks and healthcare settings. We quantified the model to represent Hlabisa subdistrict, KwaZulu-Natal, South Africa. We predicted the HIV epidemic dynamics, number on ART and program costs under the new guidelines relative to treating patients at ≤200 cells/µl for the next 30 years. During the first five years, the new WHO treatment guidelines require about 7% extra annual investments, whereas 28% more patients receive treatment. Furthermore, there will be a more profound impact on HIV incidence, leading to relatively less annual costs after seven years. The resulting cumulative net costs reach a break-even point after on average 16 years.

**Conclusions:**

Our study strengthens the WHO recommendation of starting ART at ≤350 cells/µl for all HIV-infected patients. Apart from the benefits associated with many life-years saved, a modest frontloading appears to lead to net savings within a limited time-horizon. This finding is robust to alternative assumptions and foreseeable changes in ART prices and effectiveness. Therefore, South Africa should aim at rapidly expanding its healthcare infrastructure to fully embrace the new WHO guidelines.

## Introduction

WHO has recently (November 2009) adopted new guidelines calling for earlier initiation of antiretroviral therapy (ART) for people infected with HIV [1]. Under the old guidelines, patients with a CD4+ cell count of ≤200 cells/µl were eligible to initiate ART. The revised guidelines call for ART initiation when CD4+ cell counts fall to ≤350 cells/µl. In April 2010 [2], South Africa adopted the new WHO treatment guidelines for pregnant women and for patients with TB co-infection, but decided at this time that the country could not expand the eligibility to all patients as this would overburden the healthcare infrastructure. For non-pregnant and non-TB HIV infected patients, the old strategy of ART at CD4+ cell counts ≤200 cells/µl remains [3].

Little is known about what impact the new WHO treatment guidelines will have on HIV epidemic dynamics and costs, especially in the long run. On the one hand ART reduces mortality and morbidity of the individual [4]–[6], and may reduce transmission of HIV through the accompanying reduction in viral load and thus infectivity [7]–[9]. On the other hand, longer survival of HIV patients increases the duration of infectiousness. While initial costs of adopting the new guidelines will be greater because of the increased number of people now eligible for treatment, in the long run costs may be saved because of the reduced number of new infections. Moreover, the initial costs per patient are generally lower when treatment is initiated earlier as patients require less additional care, e.g. for treatment of opportunistic infections [10], [11].

With an estimated 5.7 million people living with HIV and an overall HIV prevalence of 18% in adults, South Africa is home to the largest population living with HIV in the world [12]. Within South Africa, KwaZulu-Natal (KZN) is the most heavily affected area, with prevalence rates in 2004 of about 24% for the adult population, peaking at 51% in women aged 25–29 years [13], [14]. The Africa Centre Demographic Information System (ACDIS) contains high quality data on demography, sexual behavior, and HIV status of about 85,000 people in the largely rural Umkhanyakunde District of KZN [15]. ART rollout in this area has expanded dramatically in the past years [16], with about 7,500 people initiating ART by end 2008 [17], and a substantial decline in HIV-related and overall mortality [18].

In this paper we estimate the long-term impact of the full WHO guidelines on the dynamics of the HIV epidemic and healthcare costs in the Hlabisa subdistrict of Umkhanyakunde in KZN, South Africa. We use STDSIM, an established microsimulation model that simulates the spread of HIV and other STIs in a population of individuals interacting through a network of sexual relationships [19], [20]. We quantify the potential net costs and life-years saved due to the new WHO guidelines compared to treating patients at ≤200 cells/µl.

## Methods

### Quantification of the model

We used data from ACDIS [15] and Hlabisa Treatment and Care Programme [16] to represent demography, sexual risk behavior, and the ART rollout in the Hlabisa subdistrict. A detailed description of the model, quantifications, and data used can be found in the *Supplementary Material S1*.

In the model, HIV patients are put on ART when they seek care and their CD4+ cell counts are at a given threshold (≤200 or ≤350 cells/µl). We assumed ART to decrease infectivity by 92% [7], [21], [22]. The survival on ART was assumed to be three times the ART naïve survival [6]. Health seeking behavior was fitted such that the modeled CD4+ cell count at the first test accurately reflects data from the Hlabisa Treatment and Care Programme [16]. We assumed a dropout rate (proportion of patients on ART stopping treatment permanently) of 1.27% per year, reflecting 5% of patients lost to follow-up after four years, as reported by Houlihan *et al* [16]. The ART component of the model is illustrated in Supporting Figure 1 in Supplementary Material S1. In the model, ART is introduced in 2004, and rolled-out in accordance with the actual rollout among the 17 primary care clinics in Hlabisa subdistrict.

**Figure 1:**
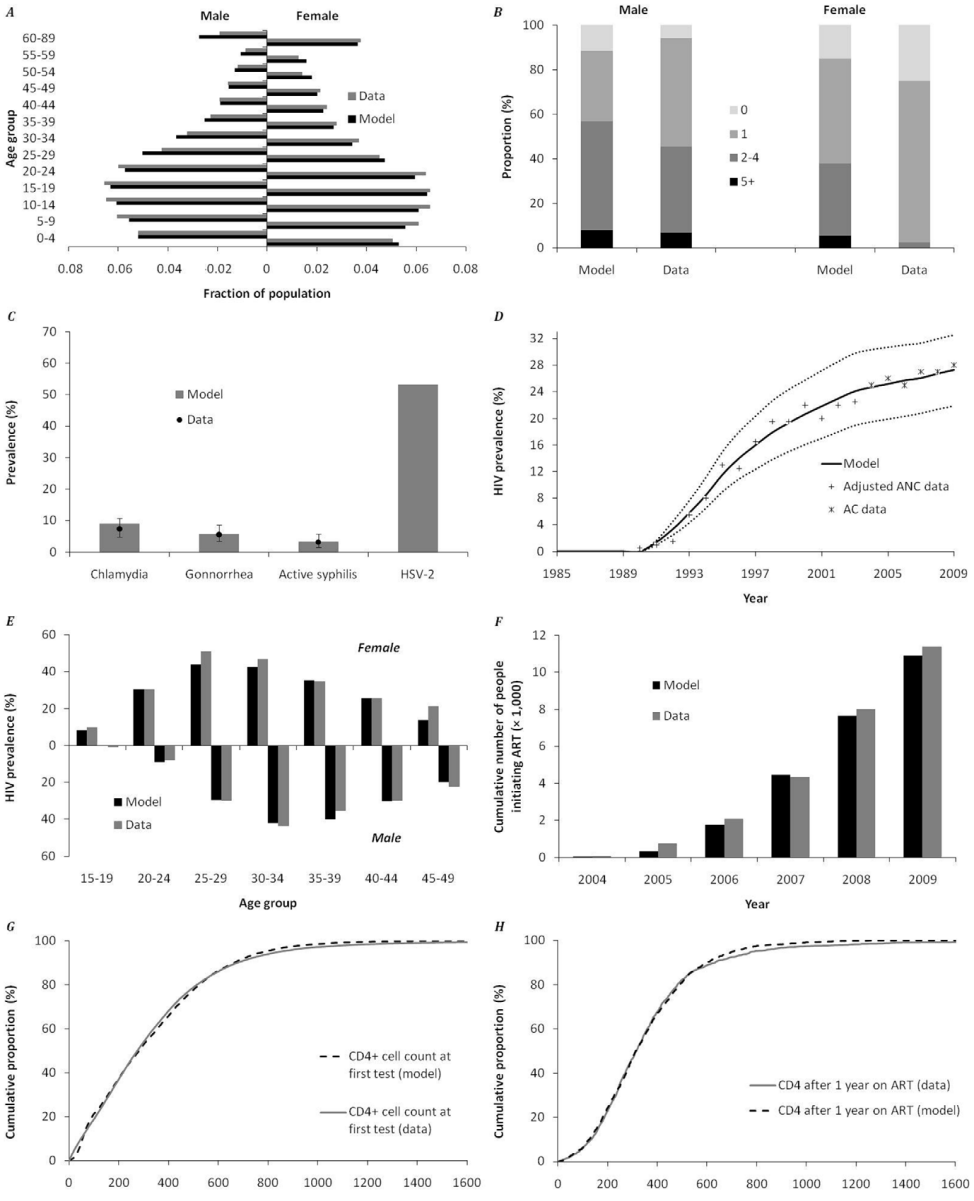
Comparison of model predictions with data of the HIV-epidemic and ART rollout in Hlabisa subdistrict of the Umkhanyakunde district in KwaZulu/Natal (KZN), South Africa. ***A.*** Modeled and actual demographic structure in 2006. Data derived from Muhwava & Nyirenda [31]; ***B.*** Total number of partners in the last 12 months in men and women aged 20–49 years in the model versus total number of reported partners derived from Todd *et al* [32] ***C.*** Modeled and observed prevalence of classic STIs in women aged 15–49 in KZN. Data derived from White *et al* [33]. ***D.*** Modeled and actual HIV epidemic in KZN. Antenatal Care (ANC) data were adjusted by applying a 1:0.6 ratio of ANC prevalence versus Africa Centre (AC) prevalence in 2004 to all data points. ANC data derived from UNAIDS [12], AC data from Bärnighausen *et al* [13]. Prevalence in 2005–2009 is from unpublished ACDIS sero-surveillance data (age specific data, adjusted for population age-structure). Dotted lines represent the predicted HIV-prevalence when assuming a 10% increase and 10% decrease in the assumed overall partner change rate (‘promiscuity factor’). The latter roughly reflects the HIV epidemic of South Africa as a whole (prevalence of 18% in 2004) ***E.*** Modeled and actual age- and sex-specific HIV prevalence in 2004. Data derived from Bärnighausen *et al* [13]; ***F.*** Cumulative number of people initiating treatment in the Hlabisa Treatment and Care Programme, model versus unpublished data [16]; ***G.*** Cumulative distribution of CD4+ cell counts at first test, model compared to data for 2007 to 2009. Data derived from the Hlabisa Treatment and Care Programme [16]. ***H.*** Cumulative distribution of CD4+ cell counts after 1 year on ART, model compared to data. Data derived from the Hlabisa Treatment and Care Programme [16].

### Costs

We analyzed costs from the perspective of the Hlabisa Treatment and Care Programme. In the absence of detailed local data, we used published data from the public sector ART programs in Cape Town, South Africa, consisting of ART costs stratified by CD4+ cell count at initiation and number of years on treatment (table 1) [10], [23].Cost values reported in these studies include costs for ART provision, treatment of opportunistic infections, outpatient visits, and inpatient days -, and consist of costs for equipment, medication, wages of healthcare personnel, logistics and infrastructure. We excluded non-healthcare costs, such as patient time and lost wages. Costs of patients on ART were stratified by CD4+ count at initiation, which reflects the fact that patients who start treatment at lower CD4+ counts are sicker and therefore in need of additional health resources (inpatient and outpatient visits, and non-ART medication). This increased medical cost of starting at a lower CD4+ cell count is most prominent in the first year of ART, and decreases in subsequent years [10]. We assumed this difference to disappear after three years. In addition, we added a one-time pre-ART cost, reflecting the cost of care surrounding treatment initiation [10]. For patients seeking care with CD4+ cell counts between 201 and 350 cells/µl and not eligible for treatment, we included costs for HIV testing and treatment for opportunistic infections [23]. For patients testing with CD4+ cell counts of >350 cells/µl, we only assumed annual costs for CD4+ monitoring [10]. Finally, we added a one-time cost of dying (hospitalization prior to death) for all HIV-related deaths, irrespective of ART status or CD4+ count at ART initiation [10].

**Table 1:** Overview of costs input values used in this study.

CD4+ count (cells/µl) at ART initiation	Per patient annual ART costs (US$)			
	*Pre-ART*	*First year*	*Second and third year*	*Subsequent years*
0–100	495	3,664	1,435	1,095
101–200	495	3,060	1,284	1,095
201–350	495	2,304	1,095	1,095

The perspective of the Hlabisa Treatment and Care Programme was chosen. Costs are stratified by CD4+ count at antiretroviral therapy (ART) initiation, and include costs of diagnostic testing, ART provision, treatment of opportunistic infections, outpatient visits, and inpatient days. In addition, some costs were included for patients seeking care but not (yet) eligible for ART: (1) 1,165 US$ per year for patients with CD4+ counts of 201–350 cells/µl, reflecting costs of testing and treatment of opportunistic infections; and (2) 104 US$ for patients with CD4+ cell counts of >350 cells/µl reflecting costs of CD4+ cell count monitoring. Furthermore, a one-time cost of dying of 1,197 US$ was included for each HIV-related death, irrespective of being on treatment and CD4+ count at initiation.

ART costs were updated to reflect present ART price levels [24]. All other costs were standardized to January 2010 prices using South Africa's consumer price index [25]. We then converted all costs into US dollars using the average exchange rate for January 2010 of US$1 to R 7.42 [26]. Costs in future years were discounted at an annual rate of 3% [27].

### Simulations

We predicted the impact of increasing the threshold for treatment initiation to ≤350 cells/µl starting by mid 2010, versus continuing with treatment initiation at ≤200 cells/µl, on HIV epidemic dynamics, number of people on ART, and associated annual costs for adults aged 15+ in the Hlabisa Treatment and Care Program in Hlabisa subdistrict until 2040. We then calculated the cumulative net costs and cumulative number of life-years saved by starting treatment at higher CD4+ counts. To roughly compare the new WHO strategy to the current South African strategy, we also assumed a scenario that a fraction of 19% of the patients with CD4+ cell counts of 201–350 cells/µl is eligible for treatment in the ≤200 cells/µl scenario (see *Supplementary Material S1*).

To correct for the stochasticity of the model, we used the average result of 1000 runs. We also presented the results of 50 individual runs to visualize the variation in model predictions. Each run was based on about 35,000 simulated individuals. Absolute values (number of people on treatment, annual costs, and life-years saved) were multiplied by 6.5 to represent the situation of Hlabisa subdistrict, which has 228,000 inhabitants [16].

### Sensitivity analysis and scenario analysis

We performed a univariate sensitivity analysis on all key parameters. Our assumption that ART reduces infectiousness by 92% is based on the most recent evidence [7], [21], [22]. However, others have assumed a reduction of 99% [28], while less reduction has also been opted [29]. Therefore, we also ran the model using reductions of 80% and 99% respectively. Dropout rates in the Hlablisa subdistrict are relatively low (1.27% per year), likely due to the experimental nature of the area. Therefore, we also assumed a dropout rate of 10% per year, which is more representative for the rest of South Africa [30], and a lower value of 1% per year. Furthermore, we made predictions for a 10% higher and 10% lower overall partner change rates (see *Supplementary Material S1*) to reflect the results for different endemicity levels, where 10% lower leads to an HIV prevalence close to that for South Africa as a whole. All other parameters (health seeking rates, survival on ART, ART costs, costs of dying, costs of not on ART) were varied by a factor 2/3^rd^ to determine the lower bound, and 3/2^nd^ to determine the upper bound.

We also performed a multivariate sensitivity analysis on 5 parameters related to the HIV epidemic and ART (overall partner change rates, survival on ART, infectivity on ART, dropout rates, and health seeking behavior rates). For each parameter we randomly selected values from Weibull (for durations) or Beta distributions (for proportions). The average of each distribution is our point estimate, and 2.5% and 97.5% values represent the lower and upper bounds chosen in the univariate sensitivity analysis. We ran 1,000 randomly selected combinations of parameters drawn from the distributions and used the 25th and 975th value to represent the bounds of the 95% confidence interval over our main outcome.

Furthermore, we assumed 4 scenarios of foreseeable future developments that could influence ART programs: (1) development of more effective ART (99% reduction in infectiousness, and increased survival by a factor 4 relative to ART naïve HIV patients); (2) further reduction in ART prices (reduced ART costs by 20%); (3) risk compensation in response to reduced threat of HIV(condom use of 10% in casual contacts); (4) resistance development (increased ART costs by 20% due to more need of second- and third-line treatment options). All scenarios are assumed to take effect in 2015.

## Results

Our model was able to accurately simulate the demographic structure, sexual behavior dynamics, and HIV and STI prevalence in the Hlabisa subdistrict both before and after the ART rollout in 2004 (figure 1). Two differences between model and data can be seen. First, the model predicts about 25% more men in the 60+ age group than observed (figure 1A), possibly as a result of a higher background mortality rate in this group compared to the Coale-Demeney life table used. However, the contribution of this age group to the overall HIV epidemic in the area is limited, so this discrepancy will not affect our main results. Second, the reported number of recent sexual partners of women is much lower than predicted by the model (figure 1B). This is likely a result of underreporting, something that is commonly observed in studies on reported sexual risk behaviour [34]–[37]. The prevalence of classic STIs, which is often viewed as a more accurate indicator of risk behaviour, accurately fits the data for women (figure 1C). As a consequence of the good fit of the underlying demography (figure 1A), risk behavior (figure 1B) and co-factors (figure 1C), the predicted HIV prevalence is very close to that observed, both over time (within 0.3% to 0.9% between 2004 and 2009; figure 1D) and within age and sex groups (figure 1E). Furthermore, the cumulative number of people initiating treatment in the model accurately reflects the actual treatment initiation numbers observed in the program (figure 1F). Finally, the model accurately fits CD4+ cell count distributions as observed in the Hlabisa Treatment and Care Programme, both at the time of initial testing (figure 1G) and one year after initiating ART (figure 1H).

**Figure 2:**
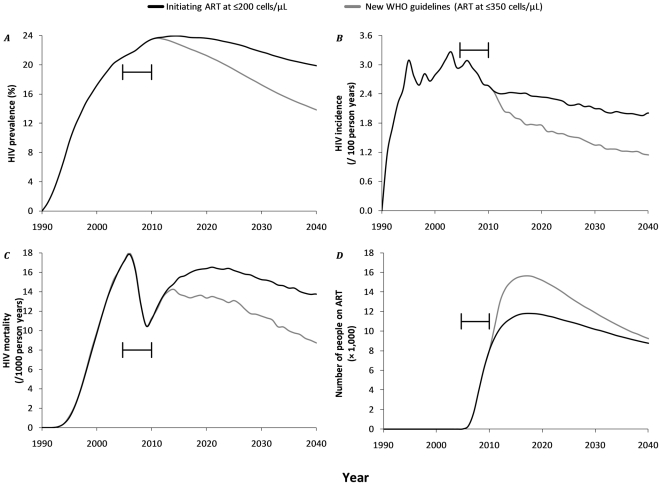
Projected impact of ART at CD4+ cell counts of ≤200 /µl (black) and the new WHO treatment guidelines of ART at CD4+ cell counts of ≤350 /µl (gray) on HIV epidemic dynamics in the Hlabisa subdistrict of the Umkhanyakunde District, KwaZulu/Natal, South Africa, 1990–2040. ***A.*** HIV prevalence; ***B.*** HIV incidence; ***C.*** HIV mortality; ***D.*** Total number of people on ART. The results reflect the average of 1000 model runs and concern adults (≥15 years). The bar indicates the timing of the initial start of ART distribution in the first clinic (end 2004) till full coverage of all 17 clinics in the area (mid 2010).

Figure 2 indicates that continued initiation of ART at CD4+ cell counts of ≤200 cells/µl will result in a modest decline of the HIV epidemic over the coming years. After peaking at 24% in 2015, HIV prevalence in adults (aged 15+) is predicted to reduce to 20% in 2040 (figure 2A). Incidence will continuously decrease from 2.6/100 person years in 2010 to 2.0/100 person years in 2040 (figure 2B). Although mortality rates were almost halved over the period 2004–2009, which is consistent with observations [18], we predict a rebound in 2010, associated with mortality in patients on ART (figure 2C). We expect that by 2018 the number of people on ART would have peaked at 11,000, up from 8,000 on treatment in 2010 (figure 2D). The new WHO guidelines of treating patients at ≤350 cells will have a more substantial effect on the epidemic, reducing prevalence to 14% and incidence to 1.5/100 person-years in 2040. Although initially the number of people on ART will peak at about 16,000 in 2018, it will rapidly decline to nearly the same number on treatment in 2040 compared to treatment at ≤200 cells/µl (figure 2D).

**Figure 3:**
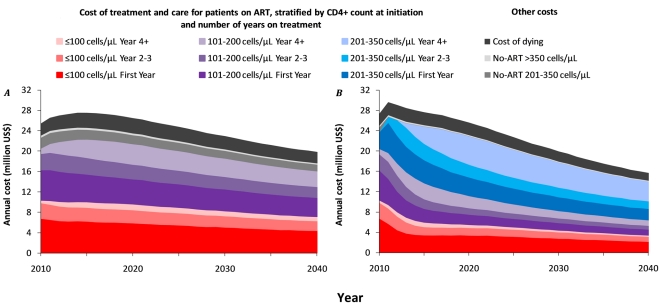
Projected cost of the ART treatment and care program in the Hlabisa subdistrict of the Umkhanyakunde District, KwaZulu/Natal, South Africa, 2010–2040. ***A.*** Annual cost when ART is initiated at ≤200 cells/µl. ***B.*** Annual cost when ART is initiated at ≤350 cells/µ. All ART costs concern adults aged 15+ and are stratified by CD4+ cell count at initiation and number of years on ART.

**Figure 4:**
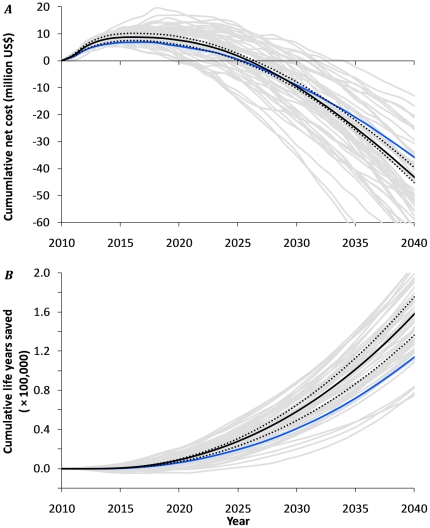
Projected net cost and life-years saved of implementing the new WHO treatment guidelines (ART at CD4+ cell counts of ≤350 /µl) versus the old treatment guidelines (ART at CD4+ cell counts of ≤200 cells/µl) in the Hlabisa subdistrict of the Umkhanyakunde District, KwaZulu/Natal, South Africa, 2010–2040. ***A.*** Cumulative net cost of treating patients at ≤350 cells/µl compared to ≤200 cells/µl. Negative values reflect net cost-savings. ***B.*** Cumulative number of life-years saved when treating patients at ≤350 cells/µl compared to ≤200 cells/µl. The black continuous line shows the average of 1000 model runs. Grey lines represent 50 individual runs to illustrate the random variation in model output. Both dotted black lines represent the results for increased and decreased levels of endemicity, when assuming a 10% higher and 10% lower overall partner change rate respectively (see also figure 1D). The blue line represents the results of treating patients at ≤350 cells/µl versus a strategy of treating patients at ≤200 cells/µl, together with 19% of patients that report with CD4+ of 201–350 cells/µl. This 19% is a crude estimation of the proportion of pregnant women and TB co-infected patients among HIV-patients with CD4+ of 201–350, who are since recently eligible to receive ART under the current South African strategy.

Figures 3A and 3B show the annual costs of treating patients at ≤200 cells/µl and ≤350 cells/µl respectively. Even though the average number of people on ART during the first five years (2011 to 2015) is predicted to be 28% higher under the new guidelines (14,000 versus 11,000, figure 2D), the average estimated annual costs are only 7% higher (US$28.6 million versus $ 26.8 million). This is because costs are mostly incurred by people initiating treatment at ≤100 cells/µl, and under the new WHO guidelines there will be significantly fewer people in this category (figure 3B in red). We predict that annual costs of treating patients at ≤350 cells/µl or ≤200 cells/µl will become equal in 2017 (figure 3), and the cumulative net costs will reach a break-even point in 2026 (figure 4A). Thereafter putting people on ART starting at CD4+ cell counts of ≤350 cells/µl will lead to net cost-savings. This break-even point is subject to stochasticity in the model and may be reached between 2020 and 2033 (gray lines in figure 4). In addition to these cost-savings, the new WHO treatment guidelines will yield about 160,000 life-years saved by 2040 (figure 4B).

These findings are not very sensitive to alternative assumptions of key parameters (table 2). Changes in cost values have the highest impact on the timing of the breakeven point, but this is limited to only 7 years. The cumulative number of life-years saved is most affected by changes in partner change rates. Similar changes in commercial sex work visits and circumcision rates had less effect on the cumulative number of life-years saved (results not shown). Multivariate sensitivity analysis shows that the uncertainty around our point estimate of the breakeven point ranges between 2023 and 2031 (table 2). Alternative scenarios of future developments regarding availability of more effective ART, further reductions in ART prices, risk compensation, and increased resistance development in the near future also have limited impact on both the timing of the breakeven point and the number of life-years saved (table 2).

**Table 2:** Sensitivity analysis and scenario analysis.

Parameter	Value	Timing of breakeven point	Cumulative number of life years saved in 2040 ( x 1000)
Baseline	-	2026	158
*Univariate sensitivity analysis*			
Partner change rates*			
Lower bound	10% decrease	2025	136
Upper bound	10% increase	2026	175
Dropout rates			
Lower bound	1% per year	2026	160
Upper bound	10% per year	2027	138
Health seeking rates**			
Lower bound	Factor 2/3	2030	142
Upper bound	Factor 3/2	2025	165
Infectivity while on ART			
Lower bound	80% reduction	2029	162
Upper bound	99% reduction	2024	158
Survival on ART			
Lower bound	Factor 2/3	2026	155
Upper bound	Factor 3/2	2026	156
ART costs***			
Lower bound	Factor 2/3	2018	N.A.
Upper bound	Factor 3/2	2033	N.A.
Cost of dying			
Lower bound	Factor 2/3	2028	N.A.
Upper bound	Factor 3/2	2025	N.A.
Cost not on ART			
Lower bound	Factor 2/3	2032	N.A.
Upper bound	Factor 3/2	2018	N.A.
Discounting			
Lower bound	1%	2026	N.A.
Upper bound	7%	2031	N.A.
*Multivariate sensitivity analysis*			
Lower bound	N.A.	2023	112
Upper bound	N.A.	2031	181
*Scenario analysis* ****			
More effective ART	infectiousness on ART reduced by 99%, survival to 4 times ART-naïve survival	2022	145
Further reduction in ART prices	Reduce ART costs by 20%	2022	N.A.
Risk compensation	Condom use reduced to 10%	2027	168
Resistance development	Increase ART costs by 20%	2027	N.A.

Timing of the breakeven point (year) and cumulative number of life-years saved (x 1000) of treating patients according to the new WHO guidelines compared to ≤200 cells/µl are shown. The breakeven point shows when cumulative net cost savings will occur.

*Effects of 10% increase and decrease in partner change rates are displayed as dotted lines in figure 1D, 4A and 4B.

**Applied to all 5 health seeking rates (*r_h_*(1) to *r_h_*(5), see *Supplementary Material S1*.

***Applied to all cost values displayed in table1.

****All scenarios changes is the scenario analysis take effect in 2015.

N.A.  =  Not Affected.

When comparing the new guidelines with the scenario that 19% of HIV patients with CD4+ cell counts of 201–350 receive ART (crudely reflecting the current South African policy of providing ART to pregnant and TB co-infected HIV patients), our model explorations show there will still be a break-even point around 2026 (blue line figure 4A). The number of life-years saved by 2040 will then be about 120,000 (blue line in figure 4B).

## Discussion

We show that starting ART at ≤350 cells/µl, as recently recommended by WHO [1], will lead to only a modest increase in program costs, but significantly more patients on ART in this rural setting of KwaZulu-Natal, South Africa. Compared to ART initiation at ≤200 cells/µl, initiating ART according to the new WHO guidelines will result in cumulative net cost-savings starting around 2026. This break-even point is robust to alternative assumptions in key parameter values. In addition to net cost-savings, the new guidelines produce a substantial increase in number of life-years saved as well as a more profound decrease in HIV prevalence and incidence.

Our baseline predictions concerning the Hlablisa subdistrict could be too optimistic for South Africa as a whole, where dropout rates are higher [30], health seeking behavior is less [12], and endemicity levels are slightly lower [12]. However, the sensitivity analysis shows that these differences have a limited impact on the timing of the breakeven point and the number of life-years saved (table 2). This can be explained by the fact that we compare two scenarios (ART at ≤200 cells/µl versus ≤350 cells/µl), which are both largely affected in the same way, so that the comparison between the two remains relatively unchanged. This demonstrates that our main finding of limited initial investments with a breakeven point within a limited time horizon is generalizable to South Africa as a whole.

We realize that the comparative strategy of starting ART at ≤200 cells/µl does not fully represent the current South African policy, since very recently the country announced that pregnant women and TB patients co-infected with HIV should initiate ART at ≤350 cells/µl [2], [3]. However, our model explorations show that when including 19% (roughly the proportion of pregnant women or TB co-infected) of the patients with CD4+ between 201–350 in the ≤200 scenario will lead to the same general finding: i.e. modest initial frontloading needed to adhere to the WHO guidelines of treating all with CD4+ ≤350 cells/µl, resulting in net cost-savings around the year 2026. The initial investments of expanding the program to include all patients with CD4+ cell counts of 201–350 cells/µl are likely to be even less than predicted by our model, because especially TB co-infected patients require more additional care and thus are more expensive than other HIV-infected patients. On the other hand, the projected number of life-years saved in this comparison is somewhat overestimated since ART for pregnant women is beneficial for both the mother and her unborn child [38].

Our baseline calculations are based on the premise that assumption will not change in the future, but it is conceivable that there may be developments that would influence the epidemiological and economic impact of ART. The development of new, more effective ART and further reduced ART prices might improve the distribution and effectiveness of ART [39], while concerns exist regarding risk compensation [40] and resistance development [41]. However, the scenario analysis shows that each of these possible future developments has limited impact on both the timing of the breakeven point and the number of life years saved (table 2).

Thus, it is clear that from an economic point of view South Africa should adopt the full WHO guidelines as soon as possible, given the expected net savings within a limited time-horizon. However, there are limitations regarding infrastructural and human resources, which are already stretched under the current efforts of South Africa to provide treatment and care for HIV-infected patients [43], [44]. This was also the reason why South Africa decided not to adopt the full WHO guidelines. Infrastructural expansion may require increased funding, resulting in the postponement of the breakeven point. However, model explorations show that, when adding 10% to the overall annual costs in the first five years of treating patients according to the new WHO guidelines in order to reflect the costs for infrastructural expansion, net cost savings will occur only 6 years later (results not shown). The expected savings achieved by adopting the full WHO guidelines could be a basis to ensure sufficient resources for infrastructural development and increase the pool of health workers through task shifting, decentralization, increased training, and higher salaries [43], [45].

The effect of ART on HIV epidemic dynamics and costs has been explored in a number of other modeling studies [21], [28], [46]–[48]. However, our microsimulation model allows more accurate modeling of sexual networks, transmission dynamics and STI co-factor effects. It is striking that our model gives such a good representation of the demographic and epidemiological situation of this setting, while only three parameters (overall partner change rate and two parameters for health seeking - see *Supplementary Material S1*) were used to calibrate the model. Moreover, it is reassuring that our model predicts a stable HIV incidence over the period 2003–2007 (figure 2B), as reported by Bärnighausen *et al* [14] and the strong decline in HIV-related mortality during the first years of ART rollout (figure 2C) is consistent with Herbst *et al* [18].

In conclusion, our study provides a strong argument in favor of immediately adopting the new WHO treatment guidelines, rather than starting ART at ≤200 cells/µl, or only implementing the guidelines for specific groups. This is provided that increased efforts are undertaken to increase human resources and healthcare infrastructure. In addition to a reduction in transmission and mortality, and a substantial increase in life-years saved, cumulative net cost-savings for treating patients with CD4+ cell counts of ≤350 cells/µl will occur after about 16 years. We show that the new WHO guidelines are beneficial from a financial, epidemiological, and societal point of view, regardless of future developments, and South Africa should therefore aim at rapidly expanding its healthcare infrastructure to fully embrace the new WHO guidelines.

## Supporting Information

Supplementary Material S1 Detailed description of model and quantification.
(DOC)
