# Supplementary material for: The Impact of the New WHO Antiretroviral Treatment Guidelines on HIV Epidemic Dynamics and Cost in South Africa
Source: PLoS One. 2011 Jul 20;6(7):e21919. doi: 10.1371/journal.pone.0021919 (PMC3140490; doi:10.1371/journal.pone.0021919)

**Supplementary material to manuscript:**

***The impact of the new WHO antiretroviral treatment guidelines on HIV epidemic dynamics and cost in South Africa***

**General model structure**

We used STDSIM, a stochastic microsimulation model of the transmission and control of HIV and other STIs [1-3]. The model simulates the life course of individuals in a dynamic network of sexual contacts. Events like partnership formation or the acquisition of infections are the result of random processes, determined by probability distributions. Therefore, the results of the model are subject to stochastic variation. It is necessary to perform multiple runs and average the results to diminish the stochasticity in predictions.

The model consists of four modules: *demography*, *sexual behavior*, *transmission and natural history*, and *interventions*. The demography module implements the processes of birth, death, and migration. Processes for initiation and dissolution of sexual relationships, for mixing according to age preference, for sexual contacts within relationships and for sexual contacts between clients and sex workers are defined in the sexual behavior module. In the transmission and natural history module, transmission probabilities per sexual contact are specified for HIV and other simulated STIs. Finally, the interventions module specifies the timing and effectiveness of control measures in curbing transmission or enhancing survival. More details about the general model structure can be found in van der Ploeg *et al* [1], Korenromp *et al* [2], and Orroth *et al* [3]. The modeling of antiretroviral therapy (ART) is new with regard to these papers, and will be explained below.

**Modeling ART**

*HIV stages and CD4+ cell counts*

HIV infection is modeled in 6 consecutive stages: an acute stage (10 weeks), 2 asymptomatic stages (125 weeks each), 2 symptomatic stages (120 weeks and 80 weeks respectively), and an AIDS stage (40 weeks) (figure S1). The resulting survival time after infection, in the absence of treatment, is on average 10 years. This is consistent with observed data [4] the same as in previous STDSIM studies [3, 5], and nearly equal to the 11 years recently assumed in the modeling study by Granich *et al* [6]. Transmission probabilities are increased by a factor of 15 during the acute stage, 3 during the symptomatic stages and 7.5 during the AIDS stage, relative to the asymptomatic stages [3, 5]. The durations of the acute, asymptomatic and symptomatic stages are assumed to be exponentially distributed, whereas a Weibull distribution with a shape parameter 2 is used to describe the AIDS stage [3].

Initial CD4+ cell counts of HIV negatives are randomly drawn from a lognormal distribution with median 7.02 (equivalent to 1116 cells/µL) and a standard deviation of 0.303, which has previously been used by Granich *et al* and Williams *et al* [6, 7]. For each individual, this initial value is multiplied by *x*, the relative CD4+ cell count, which starts at 1 and continuously decreases during HIV progression (red line in figure S1). Analogous to Granich *et al* [6] and Williams *et al* [7], we assumed a rapid decline of *x* to 0.75 during the acute stage, followed by a linear decrease during the remaining stages until *x* = 0.005, after which the individual dies of AIDS [6, 7].

*ART*

At a rate *r_h_* (*i*), patients can visit a clinic and get a CD4+ cell count test. These health seeking rates can be varied per HIV stage (figure S1). When the CD4+ cell count is equal to or below a given threshold (200 or 350 cells/µL), ART is initiated, and the patient moves to the corresponding ART stage (figure S1). The durations of the HIV stages on ART are three times that of the ART-naïve HIV-infected, based on a study by Walensky *et al*, who used randomized trials and observational cohorts to assess the survival of HIV-patients on ART [8]. ART is assumed to decrease infectivity of HIV by 92%, based on a meta-analysis by Attia *et al* [9]. This value was also used in a recent modeling study by Dodd *et al* [10] and is the same as recently found by Donnell *et al* [11]. Patients stop treatment permanently at a rate *r_d_* (annual dropout rate).

**Baseline quantification of STDSIM**

The model parameters were quantified to represent the Hlabisa subdistrict of the Umkhanyakunde District in KwaZulu-Natal (KZN), South Africa [12, 13].

*Demography*

We used location-specific fertility- and migration-rates in order to fit the demographic structure of the Hlabisa subdistrict [14]. In recent years, fertility rates in the area have been declining [15, 16]. Therefore, we adjusted fertility rates in accordance with published values: 4.4 until 1992; 3.9 in 1992-1996; 3.3 in 1997-2001; and 2.8 from 2002 onwards [16]. We used age- and sex-specific in- and out-migration rates as published by the Africa Centre [14]. Background mortality rates (i.e. excluding HIV-related death) were based on Coale-Demeney life tables (table: *South 55*) [17].

*Sexual risk behavior*

We assumed an average age of sexual debut of 18 years for women and 20 for men, based on observational data [18]. The study area is characterized by significant amounts of circular migration with about 60% of the adult male population spending most nights away in urban areas, where they frequently have additional sexual partners, often including sex workers [14, 19, 20]. We therefore adopted rates of visits to sex workers used in the STDSIM quantification for Kisumu (Kenya) [3], which has an HIV prevalence similar to that in our study population: ANC prevalence 34% in Kisumu [3] versus 36% in KZN [21] in 2000. The overall partner change rate (‘promiscuity factor’), which reflects the tendency of individuals to become available to form new sexual relationships [2], was calibrated such that the model accurately reflects the trend in HIV prevalence as available from antenatal clinic (ANC) data (1990-2004) [21] and ACDIS sero-surveillance data (2004-2009) [4]. In 2004, the population prevalence in the ACDIS cohort was 0.6 (25%/41%) that of the ANC prevalence in KZN, and we therefore multiplied the ANC prevalences by this factor. The resulting fit of the HIV prevalence is illustrated in figure 1D in the main text, which also shows the predicted prevalence for a 10% increase and 10% decrease in the best-fitting overall partner change rate.

*Transmission and natural history*

We modeled the following STIs: HIV, Chancroid, Gonorrhea, Chlamydia, Syphilis, and HSV-2.

All biological parameters (transmission probabilities, co-factor effects, and natural history) are the same as in the recent STDSIM application for the *Four cities study* [3, 22], and can be found in table 1 of Orroth *et al* [3]

*Interventions*

We assumed a linear increase in the rate *r_h_* (*i*) of seeking and receiving voluntary counseling and testing for HIV, as a function of stage number *i* (*i* = 1 to 5), not including the acute stage (figure S1). We estimated an intercept and slope by fitting the predicted distribution of CD4+ cell counts during the patient‘s first test to that recorded in the Hlabisa Treatment and Care Programme [13] (intercept = 0.1 tests/year, slope = 1.1). In the model we phased in ART in accordance with the timeline of the actual rollout among the 17 clinics in Hlabisa subdistrict. Whenever a new health facility started distributing ART, we increased the number of patients seeking care by 1/17^th^. Furthermore, we assumed that clinics start at 50% capacity, and run at full capacity after 1 year. After 4 years of the ART program, 5% of the population that initiated ART was observed to be lost to follow-up [13]. Therefore, in the model we assumed an annual dropout rate *r_d_* of 1.27% and we further assumed that these patients do not initiate ART again.

We assumed an increase in condom use in casual (non-marital) and sex worker contacts in 1998 (from 0% to 10%) and 2003 (from 10% to 20%), according to KZN data [23-25]. For the whole study period, we assumed that condoms were not used in steady (marital) relationships. In addition, we incorporated a slight improvement in STI treatment coverage based on the introduction of syndromic treatment guidelines in 1995 [26, 27] (Men: coverage of treatment for chlamydia- and gonorrhea-symptoms from 20% to 50%, syphilis- and chancroid-symptoms from 20% to 60%; Women: coverage of treatment for chlamydia- and gonorrhea-symptoms from 15% to 40%, syphilis- and chancroid-symptoms from 15% to 50%). We used the best available estimate of the KZN circumcision rate (26% in 2003) [25].

**Additional assumptions to represent the current South African policy**

We did not explicitly model the current South African policy of putting pregnant and TB co-infected HIV patients earlier on ART, as this would lead to an undue increase of complexity in the model. However, to roughly compare the new WHO strategy to the current South African strategy, we also assumed a scenario that a fraction of the patients with CD4+ cell counts of 201-350 cells/µl is eligible for treatment. The Hlabisa Treatment and Care Programme did not record TB or pregnancy status of patients with CD4+ cell counts of >200 cells/µl. However, the proportion of pregnant women among those initiating treatment at ≤200 cells/µl was about 4%. It is likely that the proportion of pregnant women in the group with CD4+ cell counts of 201-350 cells/µl is considerably higher [28], say 10%. The prevalence of TB among HIV patients in this area is high (25%), but these mainly concern patients with low CD4+ cell counts [29]. When assuming 10% of HIV patients testing with CD4+ cell counts of 201-350 having TB, this results in 19% of the 201-350 group being eligible for ART under the current South African policy, either for being pregnant or having TB co-infection.

**References**

1. van der Ploeg CPB, Van Vliet C, De Vlas SJ, Ndinya-Achola JO, Fransen L, et al. (1998) STDSIM: A microsimulation model for decision support in STD control. Interfaces 28: 84-100.

2. Korenromp EL, Van Vliet C, Bakker R, De Vlas SJ, Habbema JDF. (2000) HIV spread and partnership reduction for different patterns of sexual behavior – a study with the microsimulation model STDSIM. Math Pop Studies 8: 135-73.

3. Orroth KK, Freeman EE, Bakker R, Buve A, Glynn JR, et al. (2007) Understanding the differences between contrasting HIV epidemics in east and West Africa: results from a simulation model of the Four Cities Study. Sex Transm Infect 83 Suppl 1: i5-16.

4. Morgan D, Mahe C, Mayanja B, Okongo JM, Lubega R, et al. (2002) HIV-1 infection in rural Africa: is there a difference in median time to AIDS and survival compared with that in industrialized countries? AIDS 16: 597-603.

5. White RG, Orroth KK, Glynn JR, Freeman EE, Bakker R, et al. (2008) Treating curable sexually transmitted infections to prevent HIV in Africa: still an effective control strategy? J Acquir Immune Defic Syndr 47: 346-353.

6. Granich RM, Gilks CF, Dye C, De Cock KM, Williams BG. (2009) Universal voluntary HIV testing with immediate antiretroviral therapy as a strategy for elimination of HIV transmission: a mathematical model. Lancet 373: 48-57.

7. Williams BG, Korenromp EL, Gouws E, Schmid GP, Auvert B, et al. (2006) HIV infection, antiretroviral therapy, and CD4+ cell count distributions in African populations. J Infect Dis 194: 1450-1458.

8. Walensky RP, Wolf LL, Wood R, Fofana MO, Freedberg KA, et al. (2009) When to start antiretroviral therapy in resource-limited settings. Ann Intern Med 151: 157-166.

9. Attia S, Egger M, Muller M, Zwahlen M, Low N. (2009) Sexual transmission of HIV according to viral load and antiretroviral therapy: systematic review and meta-analysis. AIDS 23: 1397-1404.

10. Dodd PJ, Garnett GP, Hallett TB. (2010) Examining the promise of HIV elimination by 'test and treat' in hyperendemic settings. AIDS 24: 729-735.

11. Donnell D, Baeten J, Kiarie J, Thomas K, Stevens W, et al. (2010) Heterosexual HIV-1 transmission after initiation of antiretroviral therapy: a prospective cohort analysis. Lancet 375: 2092-2098.

12. Tanser F, Hosegood V, Bärnighausen T, Herbst K, Nyirenda M, et al. (2009) Cohort Profile: Africa Centre Demographic Information System (ACDIS) and population-based HIV survey. Int J Epidemiol 37: 956-962.

13. Houlihan CF, Bland R, Mutevedzi P, Lessels RJ, Ndirangu J, et al. (2010) Cohort Profile: Hlabisa HIV treatment and care programme. Int J Epidemiol (published ahead of print: Feb 12).

14. Muhwava W, Nyirenda M. Demographic and socio-economic trends in the ACDIS, monograph No 2. Mtubatuba, South Africa: Africa Centre for Health and Population Studies; 2007 (accessed April 30, 2010, at http://www.africacentre.ac.za/Default.aspx?tabid=105).

15. Camlin CS, Garenne M, Moultrie TA. (2004) Fertility trend and pattern in a rural area of South Africa in the context of HIV/AIDS. Afr J Reprod Health 8: 38-54.

16. Moultrie TA, Hosegood V, McGrath N, Hill C, Herbst K, et al. (2008) Refining the criteria for stalled fertility declines: an application to rural KwaZulu-Natal, South Africa, 1990-2005. Stud Fam Plann 39: 39-48.

17. Coale AJ, Demeny P. Regional Model Life Tables and Stable Populations. New York: Academic Press; 1983.

18. McGrath N, Nyirenda M, Hosegood V, Newell ML. (2009) Age at first sex in rural South Africa. Sex Transm Infect 85 Suppl 1: i49-55.

19. Lurie MN, Williams BG, Zuma K, Mkaya-Mwamburi D, Garnett GP, et al. (2003) Who infects whom? HIV-1 concordance and discordance among migrant and non-migrant couples in South Africa. AIDS 17: 2245-2252.

20. Campbell C. (1997) Migrancy, masculine identities and AIDS: the psychosocial context of HIV transmission on the South African gold mines. Soc Sci Med 45: 273-281.

21. UNAIDS. Epidemiological Fact Sheet on HIV and AIDS South Africa. Geneva: UNAIDS; 2008. (accessed April 30, 2010, at http://www.unaids.org/en/CountryResponses/Countries/south_africa.asp)

22. Carael M, Holmes KK. (2001) Dynamics of HIV epidemics in sub-Saharan Africa: introduction. AIDS 15 Suppl 4: S1-4.

23. Demographic and Health Survey of South Africa. Pretoria: Department of health; 1998. (accessed April 30, 2010, at http://www.doh.gov.za/facts/1998/sadhs98/)

24. Colvin M, Abdool Karim SS, Connolly C, Hoosen AA, Ntuli N. (1998) HIV infection and asymptomatic sexually transmitted infections in a rural South African community. Int J STD AIDS 9: 548-550.

25. Demographic and Health Survey of South Africa. Pretoria: Department of Health; 2003.

(accessed April 30, 2010, at http://www.measuredhs.com/countries/country_main.cfm?ctry_id=55&c=South Africa)

26. Wilkinson D, Connolly AM, Harrison A, Lurie M, Karim SS. (1998) Sexually transmitted disease syndromes in rural South Africa. Results from health facility surveillance. Sex Transm Dis 25: 20-23.

27. White RG, Moodley P, McGrath N, Hosegood V, Zaba B, et al. (2008) Low effectiveness of syndromic treatment services for curable sexually transmitted infections in rural South Africa. Sex Transm Infect 84: 528-534.

28. Rollins NC, Coovadia HM, Bland RM, Coutsoudis A, Bennish ML, et al. (2007) Pregnancy outcomes in HIV-infected and uninfected women in rural and urban South Africa. J Acquir Immune Defic Syndr 44: 321-8.

29. Houlihan CF, Mutevedzi PC, Lessells RJ, Cooke GS, Tanser FC, et al. (2010) The tuberculosis challenge in a rural South African HIV programme. BMC Infect Dis 10: 23.

**Captions to figures**

**Figure S1. Model representation of HIV stages, CD4+ cell counts and ART.** Each box represents an HIV stage with corresponding average duration in weeks. The red line in the boxes represents the relative CD4+ cell count *x*, which overall declines from 1 (top of the box) to 0.005 (close to bottom of the box). After infection patients enter in the acute phase of the ART naïve HIV infection and progress through five stages after which they die (*Death*). Parameters *r_h_* (1) to *r_h_* (5) are rates of successful health seeking behavior of HIV infected individuals in the corresponding ART-naïve stages. Health seeking behavior rates are assumed to increase linearly with stage number. Patients initiate ART when their CD4+ cell count is below a certain threshold (i.e. 200 cells/µL or 350 cells/µL). Parameter *r_d_* is the dropout rate of patients on ART. The dashed vertical lines in the ART naïve boxes represent the average duration until HIV infected individuals reach CD4+ cell counts of 350 cells/µL (left) and 200 cells/µL (right), respectively.


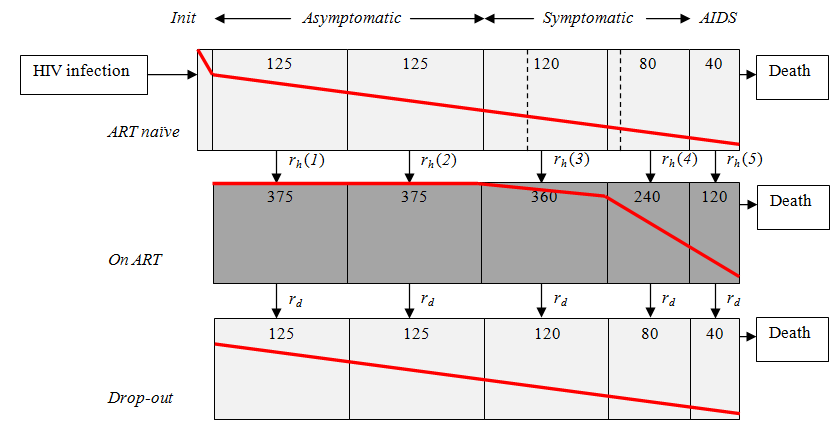

Supplement: Supplementary Material S1 — Detailed description of model and quantification. (DOC) [file pone.0021919.s001.doc]
